# Ferroelectric and magnetic properties of Nd-doped Bi_4 − *x*_FeTi_3_O_12_ nanoparticles prepared through the egg-white method

**DOI:** 10.1186/1556-276X-7-511

**Published:** 2012-09-18

**Authors:** Khalid Mujasam Batoo, Joselito Puzan Labis, Ritu Sharma, Mahavir Singh

**Affiliations:** 1 King Abdullah Institute for Nanotechnology, King Saud University, P.O. Box 2460, Riyadh, 1151, Saudi Arabia; 2 Department of Physics, Himachal Pradesh University, Summer Hill, Shimla, 171001, India

**Keywords:** Nanoparticles, Multiferroic, Dielectric constant, dc magnetization

## Abstract

Multiferroic behavior of Bi_4 − *x*_Nd_*x*_FeTi_3_O_12_ (0.0 ≤ × ≤ 0.25, × = 0.05) ceramic nanoparticles prepared through the egg-white method was investigated. The dielectric properties of the samples show normal behavior and are explained in the light of space charge polarization. Room temperature polarization-electric field (*P-E*) curves show that the samples are not saturated with maximum remanence polarization, *P*_r_ *=* 0.110 μC/cm^2^, and a relatively low coercive field, *E*_c_ = of 7.918 kV/cm, at an applied field of 1 kV/cm was observed for 5% Nd doping. The room temperature M-H hysteresis curve shows that the samples exhibit intrinsic antiferromagnetism with a weak ferromagnetism. These properties entitle the grown nanoparticles of BNFT as one of the few multiferroic materials that exhibit decent magnetization and electric polarization.

## Background

Recently, there has been an extensive study in the direction of search for the materials possessing magnetic as well as the ferroelectric properties because of the richness of physics involved in the system as well as their potential applications in memory devices and functional sensors [1-6]. These materials exhibit phenomena such as the control of electrical polarization by the application of an external magnetic field or vice versa, providing an additional degree of freedom for the design of new devices. Materials can be considered as multiferroic where ferroelectricity and ferromagnetism make mutually exclusive group [3] with the interaction of electric and magnetoelectric effects [4,7] and the effect of mutual influence of the polarization and magnetization. These phenomena are of practical interest for microelectronics, magnetic memories, sensors, and nonvolatile ferroelectric random access memory applications [3,8,9]. In order to be used as microelectronics and sensor techniques, magnetoelectric materials should satisfy this criterion: the magnetic and electric ordering temperature must exceed the room temperature. However, up to now, multiferroic materials for room temperature applications are very few [10]. Taking into account the recent literature, most of the published articles are referring to perovskite structures as potential multiferroics [3,11]. However, only BiFeO_3_ has proved multiferroic properties at room temperature [7,12], and its complex properties are not yet well understood. Numerous studies for the search of multiferrioc properties of BiFeO_3_ system substituted with PbTiO_3_, La, Co, Nd, and Gd have been carried out in order to improve its ferroelectric and ferromagnetic properties [13,14]. In the light of continued search for the multiferrioc materials, the substitution of Nd was used to enhance the electrical resistivity of Ba_4_Ti_3_FeO_12_ (BNTF) system. This paper reports the synthesization of Nd-substituted nanomaterials through the egg-white method and their dielectric, ferroelectric, and magnetic studies.

## Methods

### Material preparation

Nanoparticles of BNTF were prepared through egg-white method. The starting materials Bi(NO_3_)_3_·5H_2_O, Nd(NO_3_)_3_·6H_2_O, TiCl_3_, and FeCl_3_ were mixed together in proper stoichiometric proportions. Extracted egg white (60 ml), dissolved in 40 ml of double distilled water through vigorous stirring, was added to the metal mixture at room temperature. After constant stirring for 30 min, the resultant sol–gel was evaporated at 80°C until a dry precursor was obtained. The dried precursor was sintered at 700°C for 10 h. The final material obtained was ground for 1 h using mortar and pestle.

The powder samples obtained were characterized for structural phase and nanosize formation using PANalytical X'Pert Pro X-ray diffractometer (The Netherlands) with Cu Kα (*λ* = 1.54 Å) in the range of 20° to 80° with a sweeping rate of 2°/min.

The microstructural and morphological analysis of the samples were carried out using a field emission scanning electron microscope (FESEM, JSM 7600 F, JEOL Ltd., Akishima, Tokyo, Japan) and field emission transmission electron microscope (HRTEM, JEOL 2010 F, JEOL Ltd.) with the energy dispersive X-ray (EDX) facility attached.

For electrical measurements, a fixed amount of powder sample was taken, and a few drops of PVA were added to it. The mixture was left over night, dried at room temperature, and pressed into disc-shaped pellets (12 mm × 12 mm) with the help of hydraulic press. The pallets were heated at 500°C for 1 h, and silver paste coating was applied on opposite flat faces of the pallets to make parallel plate capacitor geometry. The dielectric measurements were performed in the frequency range 1 kHz to 1 MHz using Wayne Kerr 6500B impedance analyzer (Wayne Kerr Electronics Ltd., Woburn, MA, USA). The polarization versus electric field hysteresis measurements were carried out at 1 kV/cm field using P-E loop tracer of Marine India, New Delhi, India. Room temperature magnetic hysteresis measurements were carried out using Lake Shore VSM (Lake Shore Croyotronics Inc., OH, USA) with a field of 20 kOe.

## Results and discussion

### Structural and morphological studies

**Figure 1:**
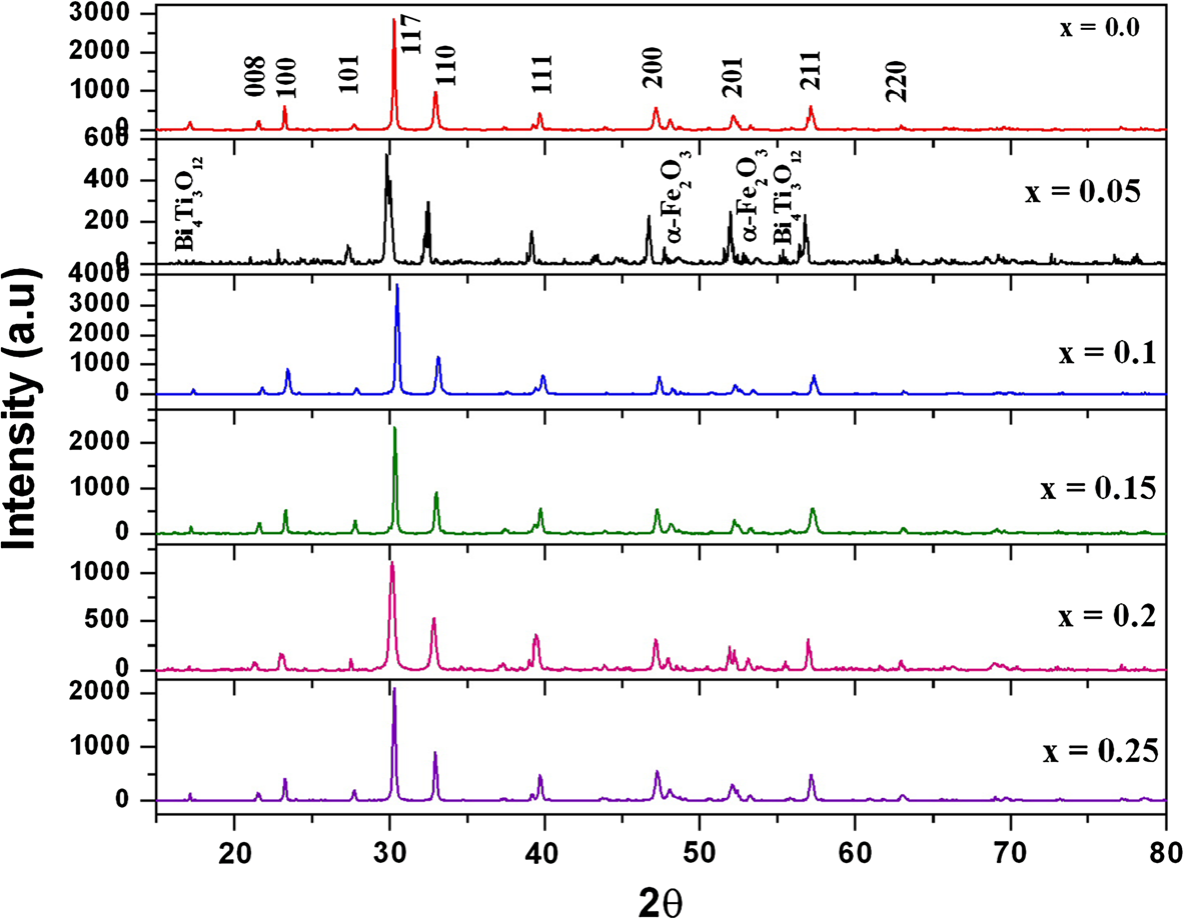
**XRD pattern for Bi**_**4 −*x***_**Nd**_***x***_**Ti**_**3**_**FeO**_**12**_**(0.0 ≤ × ≤ 0.25, × = 0.05) nanoparticles.**

**Figure 2:**
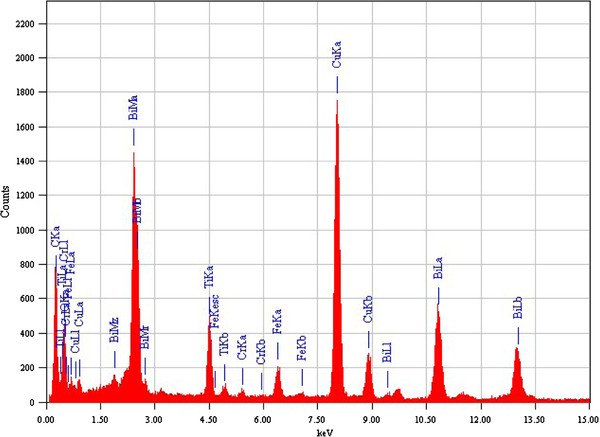
**EDX pattern for the pure (*x*** **= 0.0) composition.**

**Figure 3:**
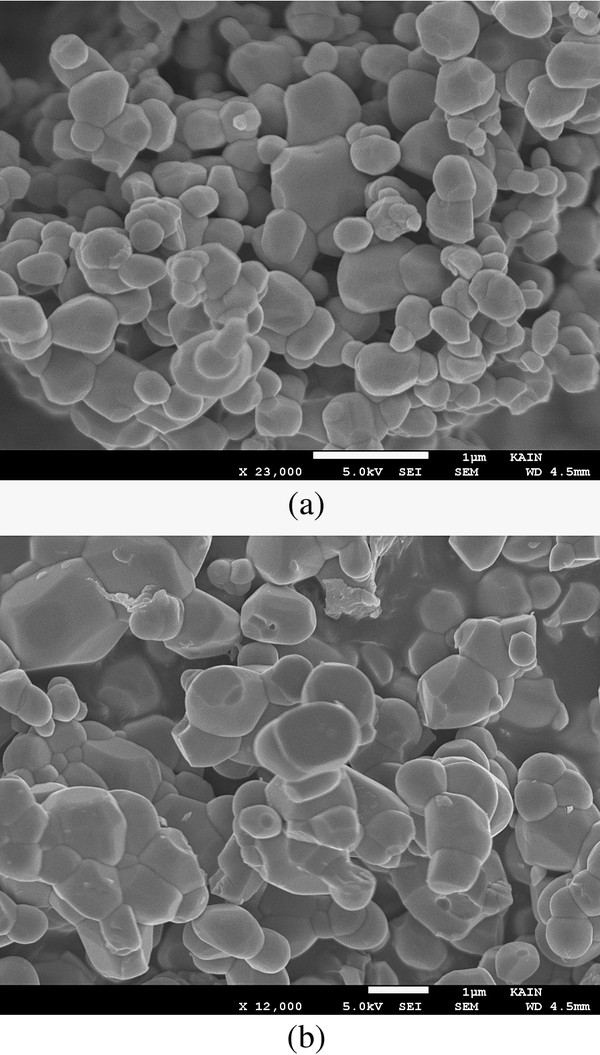
**FE-SEM images of the Bi**_**4 −*x***_**Nd**_***x***_**FeTi**_**3**_**O**_**12**_**for*x*** **= 0.0 (a) and 0.05 (b) compositions.**

**Figure 4:**
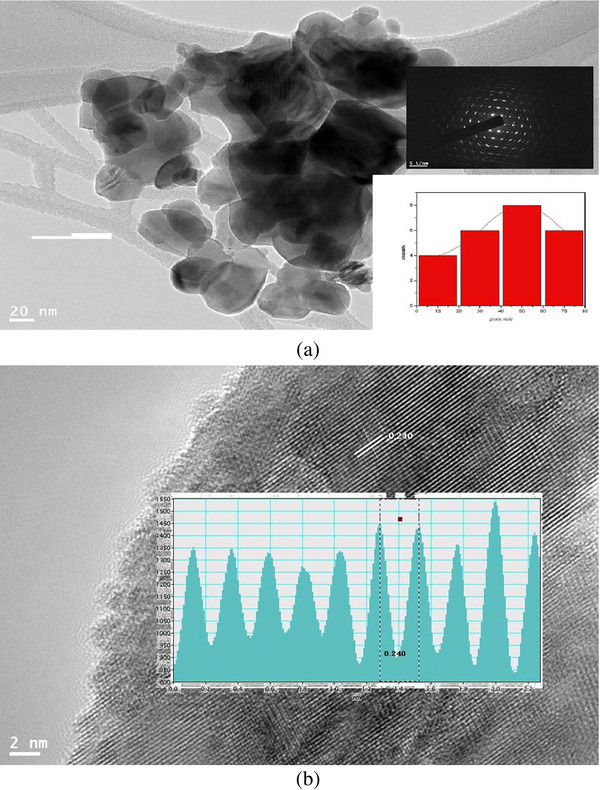
**FE-TEM micrograph and lattice planes.** (**a**) FE-TEM micrograph. Lower inset, grain size distribution; upper inset, selective area electron diffraction pattern. (**b**) Lattice planes. Inset, lattice plane spacing for the composition *x* = 0.05.

**Figure 5:**
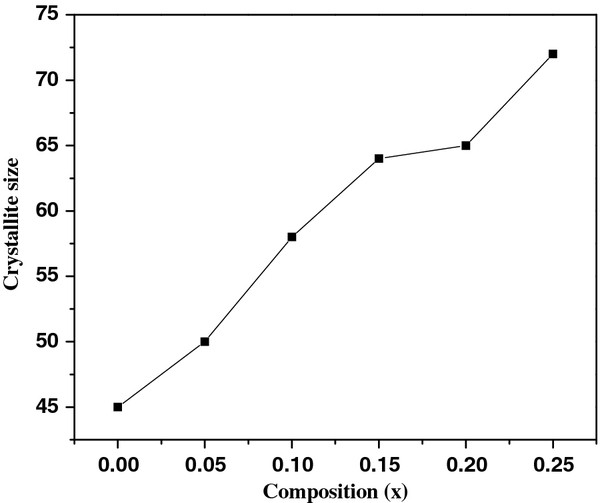
Grain size distribution with composition.

The powder samples of BNTF were characterized for structural and phase analysis through X-ray diffraction shown in Figure 1. The XRD patterns for annealed samples reveal the characteristic well-crystallized pattern with a few signatures of secondary phase corresponding to pure Bi_4_Ti_3_O_12_ compound and alpha-Fe_2_O_3_. Figure 2 shows the EDX pattern of the pure sample confirming the chemical formation of the polycrystalline BNTF nanoparticles. Figure 3a,b shows the FE-SEM microstructure of the fracture surfaces of pristine and 5% doped Nd sample. Interestingly, with Nd doping, the densification is promoted in the grown nanoparticles. Figure 4a shows the FE-TEM micrograph with inset showing the average grain size plot and selective area electron diffraction pattern for the composition *x* = 0.0. The micrograph shows irregular-shaped highly agglomerated nanoparticles with an average grain size of 50 nm for the composition *x* = 0.05. The average crystallite sizes calculated through FE-TEM show a broad size distribution from 50 to 72 nm as shown in Figure 5. A high crystalline order is observed in the grown nanoparticles. Figure 4b shows lattice pattern for the composition *x* = 0.05 with inset showing the *d* spacing value of 0.240 Å. The *d* value obtained collaborated well with the value obtained from X-ray diffraction pattern.

### Dielectric study

**Figure 6:**
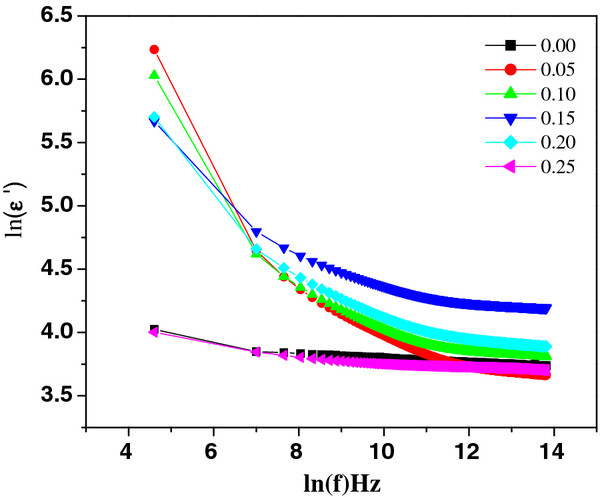
Variation of dielectric constant with frequency.

**Figure 7:**
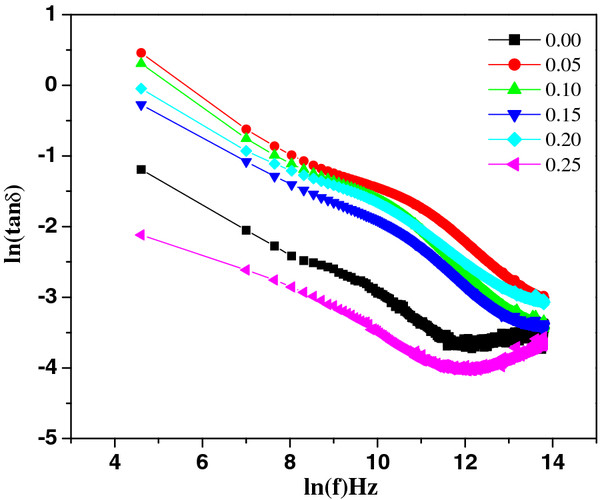
Variation of dielectric loss with frequency.

**Figure 8:**
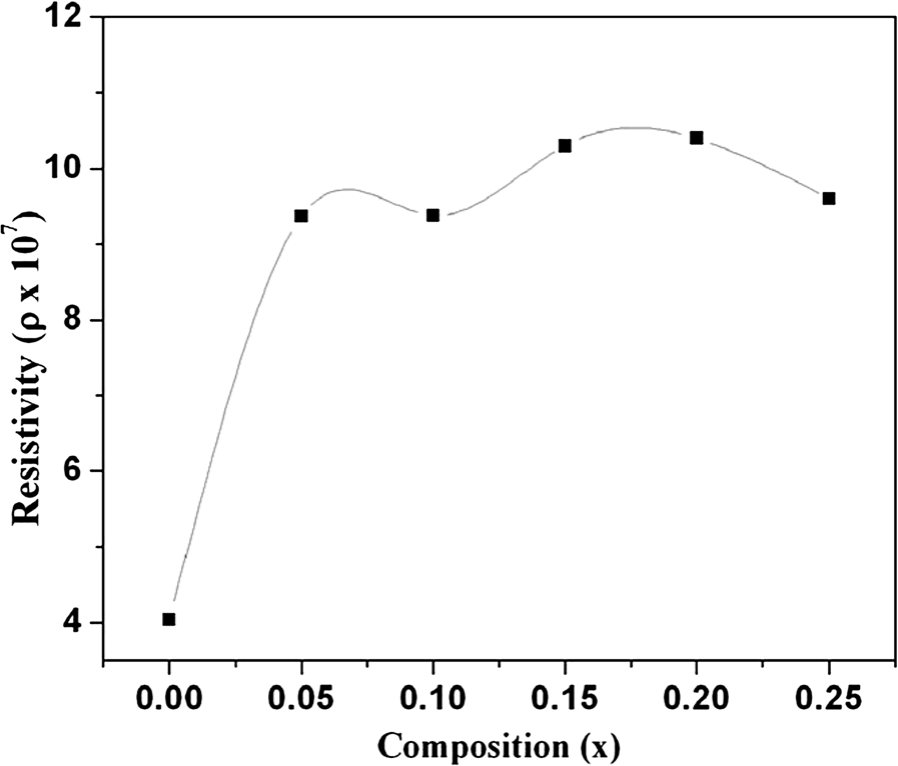
Variation of resistivity with Nd composition.

The high resistivity and low dielectric loss (tan*δ* ≈ 1.6 at 42 Hz at RT) in Nd-substituted specimens allowed the dielectric constant (*ϵ*′) to be determined, as shown in Figure 6. The room temperature dielectric constant was found 515 at 1 kHz maximum for 5% Nd concentration. The obtained dielectric constant is higher than the values of thin films (≈107) [15,16] and Nb-doped BiFeO_3_ ceramics [17] reported earlier. Both the dielectric constant and loss tangent (Figure 7) are found to decrease rapidly in low-frequency region and show frequency independent response above 22 kHz. These variations can be explained in the light of space charge polarization as discussed by Maxwell [18] and Wagner [19] and is in good agreement with Koop's phenomenological theory [20]. At low frequencies, the space charges are able to follow the frequency of the applied field, while at high frequencies, they may not have time to build up and undergo relaxation. The low loss values at higher frequencies show potential applications of these materials in high-frequency microwave devices. Moreover, the dielectric loss factor also depends on a number of factors, such as stoichiometry and structural homogeneity, which in turn, depend upon the composition and sintering temperature of the samples [21]. The room temperature resistivity measurements as a function of composition *x* are presented in Figure 8. It is seen that the resistivity of the samples increases with the increasing percentage Nd doping. The behavior may be attributed to the decreasing number of the conduction ions.

### Ferroelectric hysteresis

**Figure 9:**
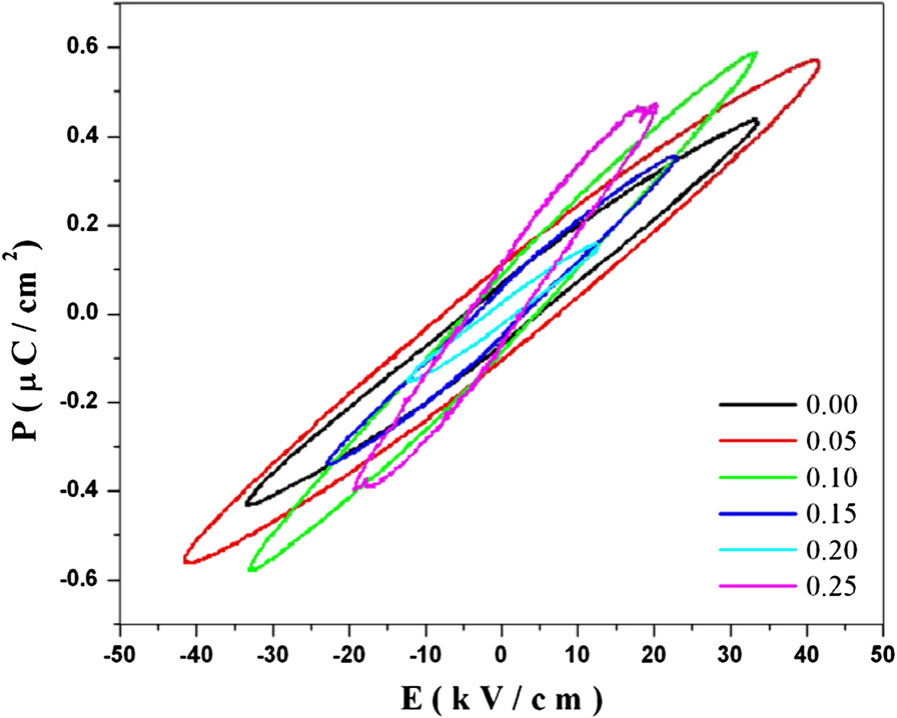
**Polarization-electric field loop for Bi**_**4 −*x***_**Nd**_***x***_**Ti**_**3**_**FeO**_**12**_**nanoparticles.**

**Figure 10:**
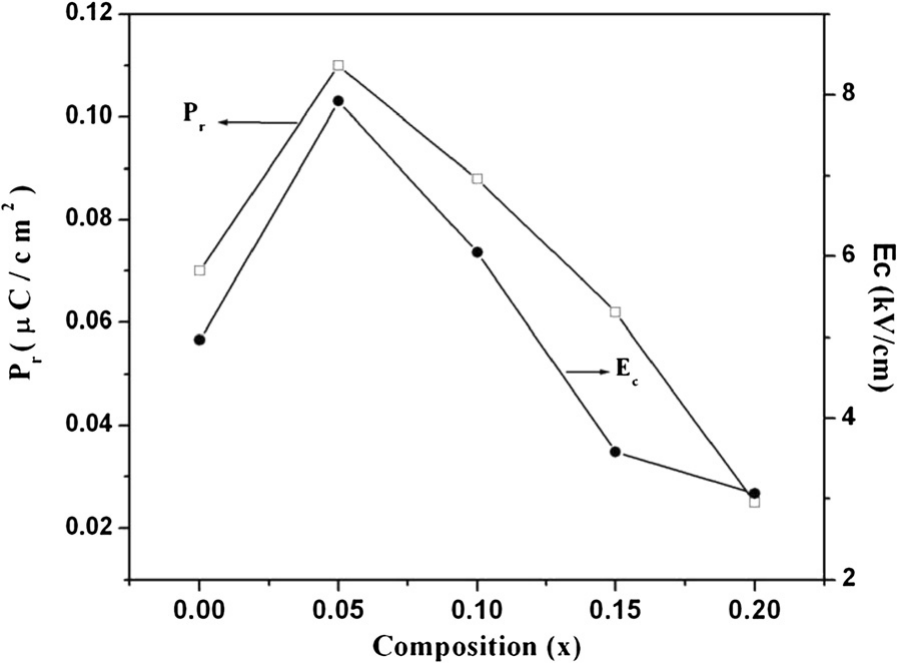
Variation of remanence polarization and coercive field with composition.

The ferroelectric hysteresis loop measurement is always hampered by the high leakage current. Because of low resistivity of the samples, it is difficult to apply high electric fields to the bulk samples. The ferroelectric polarization hysteresis loops at room temperature for Nd-doped Bi_4 − *x*_FeTi_3_O_12_ samples measured under an applied field (*E*) of about 10 kV/cm are presented in Figure 9. The loops are not really saturated and represent a partial reversal of the polarization almost elliptical-shaped [22,23]. The *P*_r_ and *E*_*c*_ values of the BNTF nanoparticles as a function of Nd composition are shown in Figure 10. The remanence polarization, *P*_r_ increases first and then decreases with an increasing vaue of *x*. The highest value of *P*_r_ = 0.110 μC/cm^2^ and a relatively low coercive field (*E*_c_) of 7.918 kV/cm were observed for 5% Nd concentration. Similar behavior in *E*_c_ is also observed where it increases first and then follows a decreasing trend with increasing Nd content [24,25]. The remnant polarization of the samples is not too high. It is well known that most magnetic materials usually have high electrical conductivity. Thus, few multiferrioc materials could exhibit the ferroelectric response properly. It is very critical for magnetic materials with high insulating resistivity to posses both ferroelectric and ferromagnetic properties simultaneously. Otherwise, an applied electric field would cause an increase in current for conducting samples rather than inducing electrical polarization.

### *M*-*H* hysteresis

**Figure 11:**
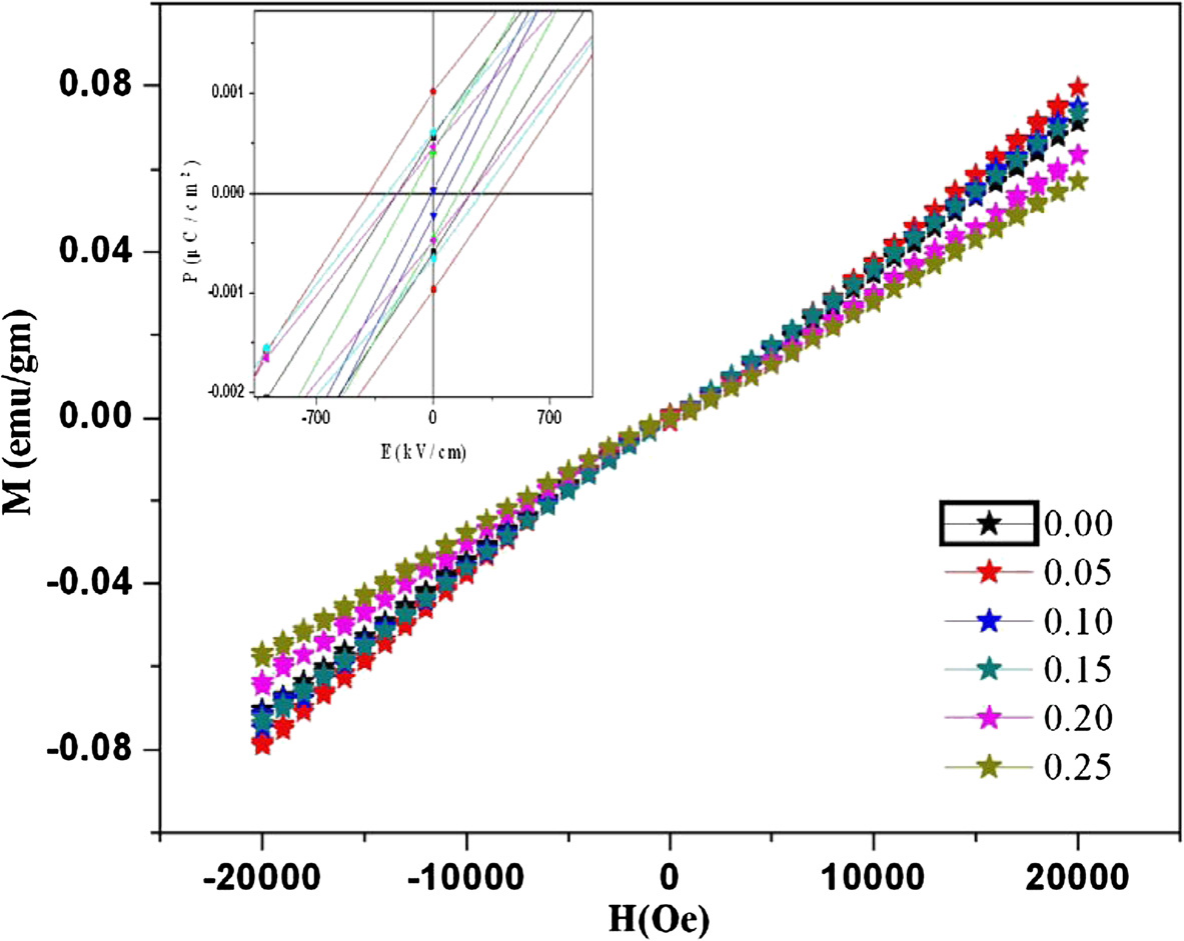
**Room temperature hysteresis magnetization.** With inset showing the magnified plot showing the ferromagnetic character of the grown nanoparticles.

Figure 11 shows the magnetization versus magnetic field (*MH*) hysteresis loops for the BNTF nanoparticles at room temperature for the maximum applied field (*H*) of 20 kOe. It is seen that all the samples show intrinsic antiferromagnetism and a weak ferromagnetism with a maximum value of remnant magnetization (*M*_r_) of 0.00107 emu/gm for sample *x* = 0.05. Various authors have reported earlier similar results [24-26]. The *M*_r_ value decreases with increasing Nd doping percentage. The substitution of Nd at Bi site may lead to the effective suppression of the spiral spin structure of BNTF, resulting in the appearance of magnetization. In order to verify and evaluate further the source of magnetism in the grown nanoparticles, room temperature Mossbauer spectroscopy measurements were tried on the present samples, but due to low percentage of Fe^57^ in the pure and doped samples, no clear Mossbauer peaks were observed.

## Conclusions

In summary, a series of nanoparticles of polycrystalline system Bi_4 − *x*_Nd_*x*_FeTi_3_O_12_ were prepared through the egg-white method to investigate the presence of multiferroic properties. The dielectric properties show normal behavior with respect to the frequency. Room temperature unsaturated multiferrioc properties were observed for the grown nanoparticles. All the samples show the intrinsic antiferromagnetism with very weak ferromagnetism. The remanence polarization (*P*_r_), and remanence magnetization (*M*_r_) values were found maximum for 5% Nd concentration. These properties entitle the grown nanoparticles of BNFT as one of the few multiferroic materials that exhibit decent magnetization and electric polarization.

## Competing interests

The authors declare that they have no competing interests.

## Authors’ contributions

The work in this paper has been mutually carried out by all authors. RS along with MS prepared and carried out the electrical and magnetic measurement of the samples. JPL carried out the FESEM, EDX, and FE-TEM measurements for the present work. KMB carried out the analysis of the data and write up of the paper. All authors read and approved the final manuscript.

## Authors’ information

KMB is working as an assistant professor in King Abdullah Institute for Nanotechnology, King Saud University, Riyadh, Saudi Arabia. He obtained his Ph.D. in Applied Physics from Aligarh Muslim University, India. His field of specialization is magnetic nanomaterials. JPL is working as an assistant professor in King Abdullah Institute for Nanotechnology, King Saud University, Riyadh, Saudi Arabia. He obtained his Ph.D. in Material Sciences from the Division of Quantum Materials Physics, Okayama University, Okayama, Japan. His field of specialization is structural and morphological studies of nanomaterials. RS is at present working as Ph.D. student in the Department of Physics, Himachal Pradesh University, Summer Hill, Shimla, India. Her field of specialization is ferrite materials. MS is working as a professor in the Department of Physics, Himachal Pradesh University, Summer Hill, Shimla, India. He obtained his Ph.D. from Himachal Pradesh University in collaboration with the Indian Institute of Technology, Kanpur, India. His field of specialization is magnetic materials.
